# The Extraction, Functionalities and Applications of Plant Polysaccharides in Fermented Foods: A Review

**DOI:** 10.3390/foods10123004

**Published:** 2021-12-04

**Authors:** Theoneste Niyigaba, Diru Liu, Jean de Dieu Habimana

**Affiliations:** 1 Institute of Nutrition and Food Hygiene, School of Public Health, Lanzhou University, Lanzhou 730000, China; theosnestus01@gmail.com; 2 Key Laboratory of Regenerative Biology, South China Institute for Stem Cell Biology and Regenerative Medicine, Guangzhou Institutes of Biomedicine and Health, Chinese Academy of Sciences, Guangzhou 510530, China; jean@gibh.ac.cn

**Keywords:** plant polysaccharides, functionality, fermented foods, applications, health benefits

## Abstract

Plant polysaccharides, as prebiotics, fat substitutes, stabilizers, thickeners, gelling agents, thickeners and emulsifiers, have been immensely studied for improving the texture, taste and stability of fermented foods. However, their biological activities in fermented foods are not yet properly addressed in the literature. This review summarizes the classification, chemical structure, extraction and purification methods of plant polysaccharides, investigates their functionalities in fermented foods, especially the biological activities and health benefits. This review may provide references for the development of innovative fermented foods containing plant polysaccharides that are beneficial to health.

## 1. Introduction

In the past few decades, chronic diseases (overweight, heart disease, diabetes, and certain cancers) caused by dietary habits, lifestyle and sociocultural factors have become severe worldwide [1,2]. Recent evidence from the Global Burden of Disease Study 2017 has shown that the global death toll due to poor diet has surpassed 10.9 million [3]. Therefore, improving dietary patterns or getting adequate nutrition is one of the most effective ways to prevent non-communicable chronic diseases and reduce mortality. Interestingly, functional foods have become increasingly prominent in the food industry owing to their potential to improve dietary efficiency by delivering essential nutrients to the human body [4]. As the global burden of disease increases and the relevance between dietary nutrition and health is more widely recognized by consumers, the unprecedented global demand for healthy and functional foods has increased. Fermented foods, including dairy (yogurts, acidified milk, creams, cheeses, and ice cream) and non-dairy products (meat, cereals, vegetables, juices, and other fruit products) [5], are ideal candidates for functional foods with excellent nutrient sources such as antioxidants, vitamins, organic acids, minerals, and other bioactive components [6]. These foods play an important role in preventing and treating chronic diseases with probiotics and/or prebiotics. Probiotics are live microorganisms gaining popularity in multiple dietary applications owing to their beneficial effects on the host’s health when used correctly [7], while prebiotics are substrates that the host bacteria preferentially use to improve their health [6]. In previous studies, the combination of probiotics and prebiotics, known as synbiotics, in food has demonstrated the ability to produce vitamin B groups such as folate and short-chain fatty acids (SCFAs) such as propionate, acetate, and butyrate, secondary bile acids, indole, and indole derivatives. These diet-dependent microbial products are not only essential for the digestion, absorption, and storage of food substrates, but also for the neural and immune system development, especially with regard to preventing neural tube defects, regulating energy expenditure, glucose and lipids metabolism, immunity, and inflammation [8,9].

Notably, prebiotics’ global demand has been estimated at over USD 8.95 billion in 2020, with the global prebiotics market forecast to grow at a compound annual growth rate (CAGR) of 7% by 2030. For example, the inulin market will expand at a CAGR of 3.1%, from USD 318.3 million in 2020 to USD 382.3 million in 2026 due to the increasing demand for inulin in diverse applications such as food and beverages [10]. Unlike oligosaccharides, polysaccharides are not only used as prebiotics, they can also be applied as thickeners, emulsifiers, stabilizers, inhibitors, adhesives, gelling, water-retaining, film-forming, and continuous release agents in fermented foods (Table 1) [11]. Therefore, polysaccharides are accounting for about 10% of their total applications in food production and are increasing exponentially. The global polysaccharide and oligosaccharide markets are anticipated to expand at a CAGR of more than 5% from 2020 to 2030, reaching a value of more than USD 22 billion by then [10].

**Figure 1:**
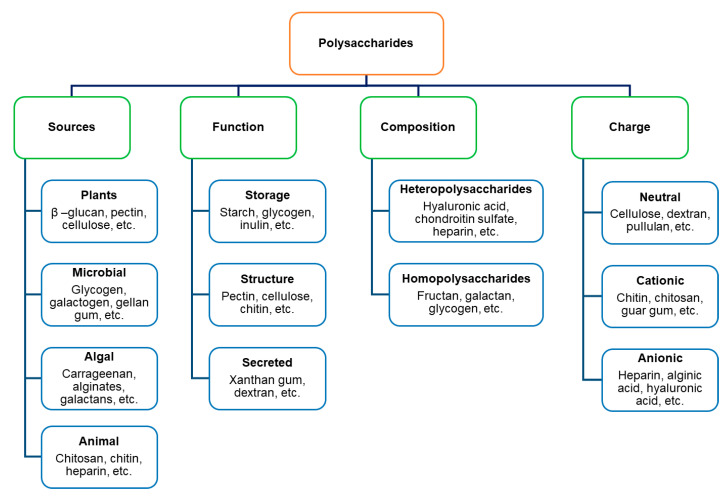
Classifications of polysaccharides.

**Figure 2:**
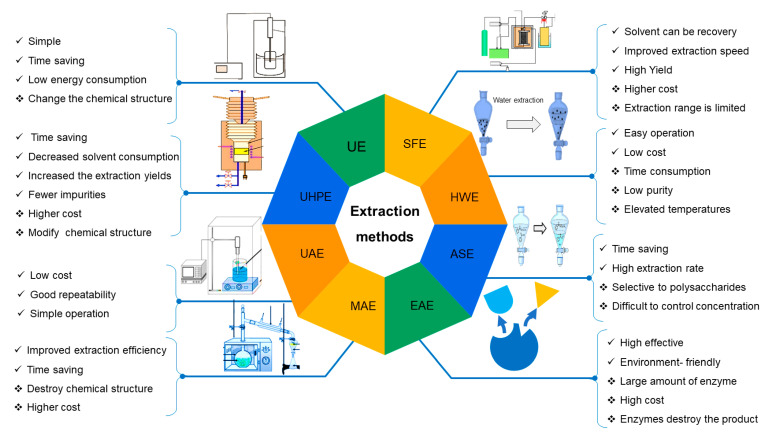
Extraction methods comparisons in terms of advantages and disadvantages. UAE, Ultrasound-assisted extraction; HWE, Hot water extraction; MAE, Microwave-assisted extraction; UE, Ultrasound extraction; EAE, Enzymatic assisted extraction; SFE, Supercritical fluid extraction; UHPE, Ultra-high-pressure extraction; ASE, Alkaline solvent extraction.

Polysaccharides are essential biopolymers formed by the natural polymerization of monosaccharides joined together by glycosidic linkages to form linear or branched-chain polysaccharides. Based on polysaccharides’ effectiveness, cost, convenience, and environmental impact, significant methods were established to extract and purify polysaccharides from different sources such as plants, animals, fungi, and microorganisms (Figure 1 and Figure 2). A variety of new natural polysaccharides from plants, indigestible by digestive enzymes found in the gastrointestinal system [12] were introduced to meet the needs of the expanding market (other than oligosaccharides), including defatted coconut residue crude polysaccharides [13], *Tragacanth* gum [14], *Sphallerocarpus gracilis* polysaccharides [12], *longan* pulp polysaccharides [15], and *loquat* leaves polysaccharides [16], contributing to novel functional foods, particularly fermented foods. It was shown that polysaccharides improve the texture, sensory, and nutritional properties of fermented foods [17] and provide a solution for consumers to improve dietary efficiency (biological activities and nutritional properties) by consuming fermented foods [18].

Although extensive reviews were conducted on the potential use of plant polysaccharides in the food matrix, emphasizing emulsification stability [19], improving texture [17] and producing colloids with functional biopolymers [20], a few of them have focused on synbiotic functional foods. For example, Tesfaye et al. [21] commented on the effects of synbiotics in dairy and non-dairy drinks on health and disease binomial approach via food. Rosa et al. [22] have focused on incorporating prebiotic components into dairy products to enhance the therapeutic effects of their consumption and the influence of their inclusion on product quality characteristics. However, in general, the importance of plant polysaccharides in fermented foods is not yet properly addressed in the literature. However, considering the significant progress made in incorporating polysaccharides into foods, it is necessary to give an overview of plant polysaccharides in fermented foods to provide a comprehensive outlook for further opportunities. Herein, this review has demonstrated a brief overview of polysaccharide classification, chemical structure, extraction and purification techniques, as well as their functional features, such as rheological properties and biological. Furthermore, the current controversy over the effects and possible health benefits of polysaccharides as prebiotics on probiotic viability in food fermentation are summarized, and the possible further research considerations on plant polysaccharides in fermented foods were discussed. Therefore, this review may provide valuable references for the development of innovative fermented foods containing plant polysaccharides that are beneficial to health.

We comprehensively searched for literature covering plant polysaccharides, focusing on extraction and purification methods, functionalities, potential applications in fermented foods, as well as health benefits. The following databases were searched for articles in English that were published until October 2021: Web of Science, Science Direct, PubMed (Medline), Scopus, and Google Scholar using the following keywords: (1) “plant polysaccharide”, “polysaccharide”, “polysaccharide from plant”, “natural polysaccharide”; (2) “extraction”, “extraction methods”, “comparison of extraction methods”; (3) “purification”, “purification methods”, “comparison of purification methods”; (4) “functionality”, “rheological properties”, “biological properties”, “bioactivity”; (5) “application”, “fat replacer”, “stabilizer”, “thickener”, “prebiotic”, “synbiotic”; (6) “dairy product”, “milk fermentation”, “yogurt cereal beverages”, “fruit and vegetables”, “juice”, “meat”; (7) “bioactive compounds”, “vitamins”, “short-chain fatty acids”, “SCFAs”; and (8) “health benefit”, “therapeutic effect”, “clinical trial”, “randomized trial”, “intake consumption”, “reduction of disease”, “treatment of disease”. The manual search approach was to combine (1) with other search keywords using “AND.” As inclusion criteria, studies evaluating natural polysaccharides derived from plants and used in fermented foods were considered. We excluded papers related to polysaccharides from other sources (animal, algal, and microbial), as well as those that discussed the polysaccharides in non-fermented foods.

**Table 1:** Plant polysaccharides used in fermented foods.

Polysaccharide	Main Sources	EM	Molecular Structure	FM	Functions	References
Cellulose	Coconut fiber Grains, fruit, vegetable	AHE, APOE, UA	β-(1→4)-d-glucopyranose, homopolysaccharides, and linear	Ice cream, sausage, cheese	Thickening agent, stabilizer, fat replacer	[23]
Pectin	Plant cell wall, vegetable, fruit	HWE, MAE, UE	α-(1→4)-d-Methoxylated galacturonic acids, Branched/coiled	Yogurt, sausage	Gelling, antimicrobial agent	[24]
β-glucan	Barley, Oat, Wheat bran	AE, ALE, EE	β-(1→4)-d-Glucose and β-(1→3)-d-glucose	Cheese	Prebiotic, fat replacer	[25,26]
Inulin	Chicory root, Jerusalem artichoke	HWE, UE	β-(1→2)-d-Fructose, linear	Yogurt, cheese	Fat replacer, texturizer, gel-forming	[27]
OP	Okra fruit	HWE, MAE, PWE	l-rhamnose (l-Rha), d-galacturonic acid (d-GalA), and (1→4)-α-GalAp-(1→2)-α-Rhap-(1→d-galactose (d-Gal)	Yogurt	Stabilizer	[28,29]
KP	Oil palm tree	AE, AE, HWE	β-glycosidic bonds	Yogurt	Prebiotic	[30,31]
DOP	*Dendrobium Officinale*	HWE, ETE	Ribose, glucose, xylose, rhamnose, arabinose, mannose, and galactolipid	Yogurt	Prebiotic	[32]
TG	*Tragacanth*	ESM	d-galactose, d-galacturonic acid, l-arabinose, l-fucose, l-rhamnose, d-xylose and d-glucose	Sausage, yogurt	Fat replacer, stabilizer, prebiotic	[14,33]

EM, Extraction methods; FM, Food matrix; OP, Okra polysaccharide; KP, Kernel polysaccharide; DOP, *Dendrobium Officinal* polysaccharide; TG, *Tragacanth* gum; AHE, Acid hydrolysis extraction; APOE, Ammonium persulphate oxidation extraction; UA, Ultrasound extraction; HWE, Hot water extraction; MAE, Microwave-assisted extraction; UE, Ultrasound extraction; AE, Acidic extraction; ALE, Alkaline extraction; EE, Enzymatic extraction; PWE, Pressurized water extraction; ETE, Ethanol extraction; ESM, Electrospinning method.

## 2. Plant Polysaccharides

### 2.1. Polysaccharides: Classification and Chemical Structure

Polysaccharides have been classified into diverse categories relying on source, structure, functions and other factors. In terms of source, they are classified into four groups: plant-derived (pectin, cellulose, starch, etc.), microbial-derived (curdlan, dextran, cellulose, etc.), animal-derived (chitosan, chitin, heparin, etc.), and algal-derived (agar, alginate, etc.) [34]. As for the composition, d-glucose is the most abundant monosaccharide in polysaccharides, followed by d-mannose, d-galactose, d-fructose, l-arabinose, l-galactose, and d-xylose. Besides, the amino sugars (d-galactosamine and d-glucosamine), their derivatives (*N*-acetylmuramic and *N*-acetylneuraminic acids), and simple sugar acids (iduronic and glucuronic acids) are also found in polysaccharides [35]. The monosaccharides in polysaccharides are linked by different glycosidic bonds, which affect the digestibility of food. For example, amylose is a linear polysaccharide consisting of d-glycose units mainly linked by α 1→4 glycosidic bonds, which does not produce insulin resistance, thus has great potential to be applied in low-fat and low-calorie foods as raw material or fat substitutes. In contrast, amylopectin, a branched polysaccharide whose main chain is connected by α 1→4 glycosidic bonds and branches are connected by α 1→6 glycosidic bonds [36,37], has a strong thickening and water holding capacity, and a higher glycemic index. Cellulose serves as a structural part of cell walls [38] and is composed of d-glucose linked by β 1→4 glycosidic bonds, which cannot be digested and assimilated by digestive enzymes existing in the body but can be decomposed and utilized by intestinal microflora. Polysaccharides can be heteropolysaccharides or homopolysaccharides based on the structure of monosaccharides. Homopolysaccharides comprise only one repeating monosaccharide unit (starch, cellulose, and inulin) [39], while heteropolysaccharides comprise the repetition of two or more distinct monosaccharides, even include additional non-carbohydrate molecules such as proteins (fibronectin, elastin, collagen) to create mucopolysaccharides, glycosaminoglycans or proteoglycans, and lipids to form glycolipids. The classification of polysaccharides based on the source, the composition of monosaccharide units, functionalities, and the charge was shown in Figure 1.

Plant polysaccharides offer diverse applications in the food and pharmaceutical sectors with distinct advantages over synthetic polymers, including availability (plants are abundant and sustainable), biocompatibility, biodegradability, low toxicity, swelling ability and water solubility [38,40,41]. The fundamental reason for the rapidly increasing interest in different plant polysaccharides is their convenience of cultivation and harvesting, which provides a continuous source of raw plant materials for polysaccharide extraction. These biopolymers are composed of multiple monosaccharide units with a high molecular weight and stable structures due to their strong intermolecular interactions [19,42]. Branching patterns, linkage, sequencing, and side-chain distributions of similar or different monosaccharide units are organized into exceedingly complex molecular structures. Furthermore, plant polysaccharides’ molecular structural properties contain numerous functional groups that may be changed or modified to generate high-quality polysaccharides [43].

### 2.2. Plant Polysaccharides Extraction and Purification

#### 2.2.1. Extraction

It is crucial to apply suitable extraction techniques that significantly influence the chemical structure, yield, consistency, and biological activities [44,45], due to its industrial application of plant polysaccharides in functional foods and pharmaceutical fields being limited by its extraction efficiency, conditions and cost. Different extraction techniques are used to produce natural polysaccharides with the basic principle of preventing polysaccharide denaturation and high efficiency [46]. The current methods for extracting plant polysaccharides include ultrasonic-assisted extraction (UAE) [47], alkaline solvent extraction (ASE) [48], enzymatic-assisted extraction (EAE), ultrasonic-assisted enzymatic extraction (UAEE) [49], microwave-assisted extraction (MAE) [50], ultrasound-microwave assisted extraction (UMAE) [51], ultra-high pressure extraction (UHPE) [38], and supercritical fluid extraction (SFE) [52], which have demonstrated a much greater extraction efficiency compared to the traditional hot water extraction (HWE) [45].

Each approach has distinct benefits and limitations regarding economic cost, material complexity, time consumption, environmental effects, energy, and extraction efficiency [50,53,54] (Figure 2). MAE is time-saving and environmentally friendly since it uses fewer solvents and energy and uses microwave radiation to increase the mass transfer of the target chemicals. However, it alters the structure of polysaccharides and is expensive [50]. The UHPE method boosts the solubility of object compounds, enabling them to permeate better into the sample matrix with reduced solvent consumption. However, this process may affect the chemical structure of polysaccharides [38]. The UAE is a fast, energy-efficient process with the least solvents and can produce high extraction rates [47]. SFE is an excellent method to extract plant polysaccharides since it is non-polluting, non-toxic and maintains polysaccharide activity. However, due to the high cost and extended extraction time, it is mostly used to extract and prepare important plant-based active components [55]. The most common method of polysaccharide extraction is a combination of hot HWE and enzymatic methods, which was shown to generate higher polysaccharide yields and purity than mono-extraction methods [56]. In addition, enzymes (cellulase, protease, pectinase, etc.) are used to hydrolyze and break down plant tissues in relatively moderate conditions (mild reaction conditions, short time, reduced chemical reagents) to accelerate polysaccharide release, even though the cost of the enzymes is relatively high [57].

Numerous researchers have compared different extraction techniques based on their extraction yield. For instance, ginger polysaccharides were extracted using HWE (100 °C, 5 h), UAE (50 °C, 50 min, 300 W), ASE (0.2 mol/L NaOH, 25 °C, 2 h), and EAE (50 °C, 90 min, 0.5%, *w*/*v*, enzyme) methods, respectively. In addition, polysaccharides from bamboo shoots were extracted using HWE (100 °C, 4 h), ASE (126 °C, 2 cycles, 22 min), UAE (49 °C, 49 min, 240 W), MAE (90 °C, 15 min, 400 W), and EAE (50 °C, 80 min, 1% complex enzyme) methods, respectively. Interestingly, the ASE method attained a maximum extraction yield of 11.38 ± 1.17% and 9.94%, respectively [50,58]. This method has gained more interest due to its ability to complete extraction in a short time with increased temperature and high pressure, resulting in increased yield [54]. Furthermore, *Fritillaria* polysaccharide and Lanzhou lily (*Lilium davidii var. unicolor*) polysaccharide were extracted by UAE, EAE (cellulase), UAEE (trypsin), and UAEE (pectinase) [54], respectively. Results revealed that the UAEE (cellulase) and UAEE (trypsin) methods achieved the greatest yields of 20.65 ± 0.78% and 9.62 ± 0.23%, respectively, compared with other enzymes. Thus, it was hypothesized that trypsin had vigorous enzymatic hydrolysis activity on the Lanzhou lily cell tissues, allowing polysaccharides to be released more quickly and efficiently in a short timeframe at a low temperature [59]. In conclusion, for different plant polysaccharides, the optimal purification methods may be different.

The polysaccharide extraction methods considerably influence the polysaccharide’s biological activities and chemical characteristics, thereby affecting their applicability [53]. Polysaccharides with vigorous emulsifying activity and large viscosity can also be employed as potential cosmetic additives, while low-viscosity polysaccharides are advantageous in producing immediate products such as butyrate [60]. Polysaccharides with low molecular weight, excellent homogeneity, and thermal stability are useful as raw materials for polysaccharide derivatization including selenylation, esterification and sulfation [60,61]. Moreover, Chen et al. [62] used HWE, UAE, EAE, and UEAE techniques to extract polysaccharides from *Crataegus pinnatifida Bunge*; the HWE technique had the highest molecular weight compared to other methods. Although all techniques had identical monosaccharide compositions, the molar percentages of monosaccharides varied. Following that, antioxidant activities revealed that the UEAE approach has the best lipid preventing ability and superoxide radical scavenging ability. Another study demonstrated the importance of choosing suitable extraction techniques to gather polysaccharides with preferred bioactivities by comparing the polysaccharides extracted from loquat leaves using HWE, UHPE, high-speed shearing homogenization extraction, MAE, UAE, UEAE, or UMAE. The results have shown that polysaccharides extracted by HWE and PWE had a significant degree of esterification and a high molecular weight, which might lead to their high (fat, cholesterol and bile acid) binding abilities. The molecular weights, total phenolic contents, and uronic acid contents had substantial inhibitory effects and antioxidant activities on in vitro α-amylase and β-glucosidase in polysaccharides extracted by MAE and UAE. Polysaccharides isolated by UMAE had lower viscosities and molecular weights, contributing to their powerful prebiotic properties [45].

#### 2.2.2. Purification

**Figure 3:**
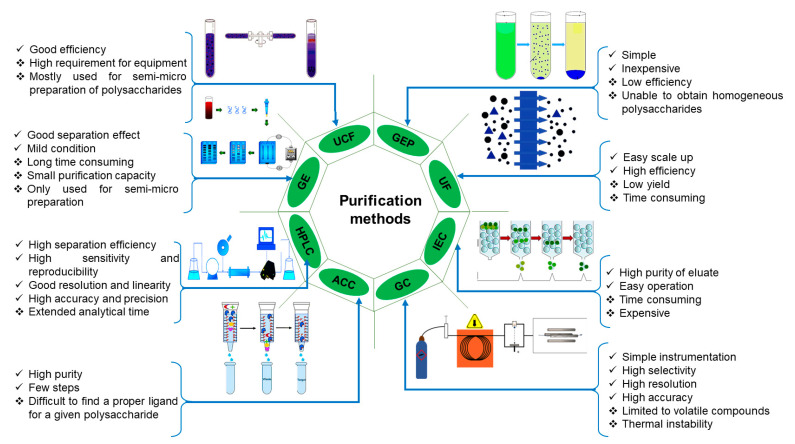
Purification methods comparisons in terms of advantages and disadvantages. UCF, Ultracentrifugation; GE, Gel electrophoresis; HPLC, High-performance liquid chromatography; ACC, Affinity column chromatography; GC, Gas chromatography; IEC, Ion exchange column chromatography; UF, Ultrafiltration; GEP, Gradient ethanol precipitation.

Polysaccharides from plants may contain many impurities, including lipids, proteins, lignin, polynucleotides, minerals and pigments. Therefore, measures have to be adopted to purify the polysaccharides before being used in food or medicine [63,64]. Natural polysaccharide products’ purity, quality, and uniformity are not comparable in molecular weight and structure due to variations in cultivation conditions, geographical location, and planting techniques [65]. As for removing extra proteins, the most common techniques for removing proteins are the Sevag method, trichloroacetic acid method, trifluoromethane and enzymolysis [57], among which the Sevag method is usually used for its simple procedure and does not readily destroy polysaccharides. However, the removal efficiency is low with significant sample loss, and it is not environmentally friendly [66]. In order to improve efficiency, the Sevag-enzyme combination is used to replace a single method. The aqueous two-phase system is a more practicable separation process that is comparatively recent due to its effectiveness in removing proteins and enriching polysaccharides at mild conditions without changing the activity of polysaccharides; thus, it is gradually displacing conventional separation methods [67,68]. Because of the lack of precision, determining the glucose content of polysaccharide products is challenging due to poor product quality [69]. The purification methods of polysaccharides can be roughly classified into three categories according to the purification mechanism and process: physical separation process (based on molecular weight and solubility); column chromatography process (based on intermolecular force); and chemical precipitation methods [46]. Membrane separation and ultracentrifugation are two methods for physical separation; the first method uses membranes with varying sources and pore sizes, such as nanofiltration, ultrafiltration, microfiltration, and reverse osmosis membranes, while the ultracentrifugation method relies on different deposition ratios. For mixed polysaccharides, membrane separation has obvious advantages according to molecular weight; for instance, membrane separation equipment is simple, has low energy consumption, and maintains a good separation effect. In addition, it does not damage polysaccharide activity, and the separation process does not introduce chemical test agents, which is pollution-free for polysaccharides themselves. However, in the process of membrane separation, the decrease in membrane osmotic flow caused by increasing concentration polarization and membrane pollution is more likely to produce membrane pollution and blockage, which is difficult to separate and purify in practical applications. Homogeneous polysaccharides may be separated by ultracentrifugation [70]. Organic reagents and salt solutions are used in chemical precipitation. Stepwise precipitation frequently uses ethanol and methanol; however, methanol is less often used because of its toxicity. The ethanol precipitation method benefits from the insolubility of polysaccharides in high-concentration alcohol. The amount of alcohol needed to precipitate polysaccharides with different properties and molecular weights is variable; therefore, the polysaccharide components can be separated from the sample [63]. Acidic polysaccharides are widely isolated using sodium, potassium, or quaternary ammonium salt solutions. Column chromatography is an excellent method for separating and purifying polysaccharides progressively using stationary and mobile phases. Based on the stationary phase filler principle, column chromatography can be split into ion-exchange column chromatography, cellulose column chromatography, gel filtration chromatography and affinity column chromatography [71]. Column chromatography separates the polysaccharides progressively using stationary and mobile phases. The most appropriate mobile and stationary phases for attaining a yield potential of the desired compound are chosen based on its physicochemical characteristics [72]. Other methods for polysaccharide purification include dialysis, high-performance liquid chromatography (HPLC), and gas–liquid chromatography [63,64]. Furthermore, the migration speed of polysaccharides influenced by a magnetic field varies depending on the polysaccharides’ charge, shape, and size properties. As a result, electrophoresis in the processing zone can be used to isolate distinct polysaccharides [46]. Figure 3 illustrates the advantages and limitations of selective purification methods.

Separation and purification of polysaccharides from the root of *Pueraria lobata* were accomplished using a Sephacryl S-100 gel filtration column and a DEAE-Cellulose 52 anion exchange column. Polysaccharide production was 0.04%, and experimental results revealed that polysaccharide had a molecular weight of 2584 Da and was a type of glucan [73]. Likewise, the black currant fruit was used to extract a new polysaccharide fraction and purified using anion-exchange Q-Sepharose FF, chromatography on macroporous resin D4006, and Sephadex G-100 columns [74]. Furthermore, owing to the difficulty of obtaining pure polysaccharides using a single technique, a mix of methods is required to accomplish effective polysaccharide purification. To this end, this procedure should also consider the sequence and scope of application of each technique. However, potential research remains challenging and essential to prepare high-purity polysaccharides on a large scale while preserving their structural similarities and bioactivity. Thus, comprehensive research of novel techniques and materials is necessary to identify the polysaccharide structural properties and assist innovative polysaccharide purification.

## 3. Functionalities and Applications of Plant Polysaccharides in Fermented Foods

### 3.1. Rheological Properties and Applications as Thickener, Stabilizer and Fat Replacer

Plant polysaccharides are distinguished by their solid structures resulting from intense covalent interactions, which make them hard to bend in response to pH and temperature changes. In food technology, polysaccharides are commonly used to alter the texture and rheology of an aqueous medium due to their strong hydrophilicity and viscosity [19] and further determine the consistency of food and enable a desired consumer pleasure for the commodity. Polysaccharides are commonly used in food in low amounts, between 1% and 3% of the formula weight. With the dissolution or diffusion in the food system, polysaccharides play different roles including thickening, gelling, stabilizing, emulsifying and moisturizing. On the other hand, these compounds also improve the fiber content and prolong the starch’s hydrolysis. In frozen foods, polysaccharides regulate the formation of ice crystals and provide strength to products undergoing continuous refrigeration cycles [75]. A previous study has shown that pectin-rich orange fiber impacted yogurt gels’ rheological, sensorial, and tribological characteristics during fermentation. Based on the gel categorization criteria, yogurts had either solid or defective gel characteristics. According to tribological findings, higher friction is caused mainly by coarse fiber yogurt’s reduced ability to immobilize water under high pressure. The lubrication properties of plain yogurt are regulated by the protein (casein) network [34]. Another study found that incorporating pectin into low-fat set yogurt minimized whey loss and enhanced firmness, texture, rheology, and optimal flavor. There was a substantial enhancement in firmness and a 16% decrease in whey losses. Moduli variations in yogurts may be attributed to the density of the protein gel matrix and hydration of the hydrocolloid activity [76].

Okra polysaccharides (OPs) exhibited standard viscoelastic and conductivity properties associated with shear-thinning. The rheological parameters of OPs revealed pseudoplastic activity and were significantly altered by concentration [77]. In this sequence, Xu et al. [42] studied the effects of four different plant polysaccharides (Okra polysaccharide, *konjac* glucomannan, sodium alginate, and apple pectin) on gelling characteristics in yogurt development. The results showed that polysaccharides improved the elasticity, water-holding ability, and firmness of yogurt. All samples showed shear-thinning activity, with visible viscosity and flexural strength. Another study discovered that replacing fat with 3.2% inulin increased the time required to reach pH = 4.8 in 11 min. A total of 3.2% inulin increased yogurt acidification rate, reduced the time required to reach pH = 4.8 by 12 min, and improved the highest storage modulus and gel firmness. In addition, spontaneous syneresis was improved by 32% [78].

Plant polysaccharides (xanthan, tara gum, guar gum, and pectin) used in dairy products as fat substitutes, stabilizers, and texturizers had a significant impact on physiochemical and sensory properties (rheology, viscosity, structure, firmness, consistency, gumminess) [79,80,81,82,83,84]. For instance, improved sensory properties distinguished yogurt with a 0.3% strong pectin concentration, a shorter acidification period, and the lowest syneresis in comparison with the control sample [79]. The addition of inulin and tragacanth gum to yogurt as stabilizers showed no significant differences in bacterial viability, color, pH, or total solids were found. Both yogurts with polysaccharides were more compact and denser than control. They displayed greater firmness and higher observable viscosity with less syneresis [80]. Polysaccharides generally can interact with milk components (water, whey proteins and casein) during fermentation and throughout storage, resulting in improved formation and stabilization of the yogurt gel network, as well as the capacity to retain the whey phase and network shrinkage. Therefore, they have the potential to produce improved yogurt that is more appealing to consumers.

As fat replacers, plant polysaccharides can also be used in cheese production to improve nutritional values. β-glucan, inulin, konjac glucomannan and other plant polysaccharides were proven to improve the textural and sensory properties (firmness, stickiness, consistency, adhesion, and gumminess, color, organic acids and better flavor) of reduced-fat Labneh cheese, Mozzarella cheese, and frescal sheep milk cheese [36,85,86]. Polysaccharides have different effects on several process stages of cheese production (structure, ripening, rennet coagulation time, taste, syneresis and moisture content), depending on their specific concentration and characteristics employed. Consequently, cheese’s nutritional and sensory characteristics (increased perceived moisture, increased breakdown rate, reduced hardness, mild and milky taste, or nutty flavor) appeal to this interaction as well [87].

Processed meat products have been characterized as refined foods that emphasize the negative aspects of saturated fat, high salt, additives, and the lack of fiber in their formulations in consumer diets [88]. Therefore, plant polysaccharides may be added to these products as thickeners, stabilizers, emulsifiers, texturizers, and gelation agents [17,89] to increase their nutritional value and help to reduce calories while improving sensory, rheological, and textural qualities. The use of mango peel pectin and inulin in dried Chinese sausage and chicken sausages has improved color to redness and yellowness as well as brighter to less reddish, respectively. In addition, their physicochemical, microbiological and sensory attributes remained unaffected [90,91]. Furthermore, the inclusion of resistant starch, oat fiber and microcrystalline cellulose (MCC) influenced sensory and physicochemical characteristics, including lactic acid bacteria (LAB), water activity, chewiness, hardness, and thiobarbituric acid reactive substance values of sausage. MCC increased LAB counts, oat fiber, and MCC exhibited antioxidant properties in a sausage. Sensory parameters such as pH values, appearance, weight loss, taste and texture, color, and proximate composition were unaffected by any dietary fibers. Resistant starch, oat fiber, and MCC were shown to produce fermented sausage with simultaneous salt (75% NaCl; 25% KCl) and fat reduction (25%) [92].

Plant polysaccharides are another possibility for meat research due to their potential effects and barely any side effects on human health, which makes them a promising candidate. Incorporating polysaccharides and probiotic strains into fermented meat products has proven valuable for creating nutritious food [92]. Developing innovative formulations with added polysaccharides and probiotics and decreased salt, nitrite/nitrate, or cholesterol content can advance meat product science to produce healthier fermented meat products.

### 3.2. Biological Activities and Applications

**Figure 4:**
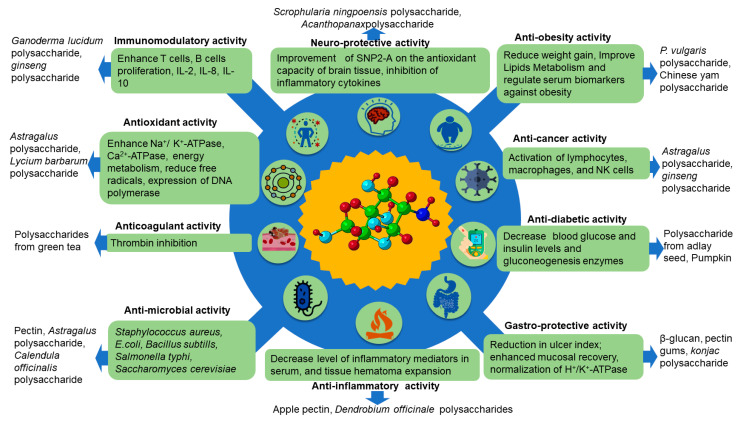
The bioactivities of plant polysaccharides and their influences on human health. SNP2-A, Single Nucleotide Polymorphisms; IL, Interleukins; NK cells, natural killer cells; Na⁺/K⁺-ATPase, Sodium-Potassium Adenosine Triphosphatase; Ca^2+^-ATPase, Calcium Adenosine Triphosphatase; H^+^/-ATPase, Hydrogen Adenosine Triphosphatase.

Polysaccharides were proven in recent research to offer a variety of biological benefits, such as anticoagulant (polysaccharide from green tea), antioxidant (*Astragalus* polysaccharide, *Lycium barbarum* polysaccharide), antibiotic (pectin, *Calendula Officinalis* polysaccharide), immunomodulatory (*Ganoderma licidium* polysaccharide, *Ginseng* polysaccharide), antidiabetic (polysaccharides from adlay and pumpkin), and anti-inflammatory (apple pectin, *Dendrobium officinale* polysaccharide) activities [93,94]. These bioactive polysaccharides derived from edible resources are safer, more effective and have fewer side effects than other sources. They are also more easily accessible and inexpensive. Thus, most bioactive polysaccharides from different plant sources constitute a significant material source for food and therapeutic applications [95]. For instance, polysaccharides isolated from psyllium seeds and husk, for example, had the greatest antioxidant and scavenging activity, with significant concentrations of 347.40 ± 1.79 μg and 362.72 ± 2.75 μg, respectively [96]. *Astragalus* polysaccharide was shown to enhance the glucose and lipid metabolism of type 2 diabetes mellitus (T2DM) rats by boosting insulin production through a protective impact on pancreatic islet beta cells [97]. Furthermore, when two polysaccharides from *Codonopsis pilosula* (named CPP1a and CPP1c) were incubated for 48 h at concentrations of 50, 200, and 400 g/mL, they significantly inhibited cell migration in human hepatocellular carcinoma (HepG2) cells, demonstrating considerable cytotoxicity. Thus, at the molecular level, altering the cell structure, initiating cell death, and arresting the cell cycle in the G2/M phase (second growth phase/mitosis phase), along with increasing the Bax/Bcl-2 (apoptosis regulator) protein expression ratio and triggering caspase-3 [98]. Figure 4 below presents further information regarding the polysaccharides’ bioactivity and biological behavior, along with typical polysaccharide examples.

#### 3.2.1. The Health Benefits of Fermented Foods Containing Plant Polysaccharides and Probiotics

##### Plant Polysaccharides as Prebiotics

Common plant-based prebiotics include fructooligosaccharides, inulin, high-performance inulin (inulin HP), xylooligosaccharides, soybean oligosaccharides, isomalto-oligosaccharides, and lactulose. While numerous plant polysaccharides including coconut residue crude polysaccharides, *Tragacanth* gum, *Sphallerocarpus gracilis* polysaccharides, *longan* pulp polysaccharides, *loquat* leaves polysaccharides [40], *dendrobium officinale* polysaccharide [42], *kernel* polysaccharide [40], and β-glucan [99] also meet the prebiotic requirements based on a set of criteria as follows: (1) absorbing resistance in the upper gastrointestinal tract; (2) selective colon fermentation through potentially beneficial bacteria; (3) selective enhancement of probiotic growth; (4) preferably induce positive impact on host health; and (5) stability in a variety of food production conditions [100].

Synbiotic, a synergistic mixture of prebiotics and probiotics, is essential for good colon health, disease prevention, and alternative methods to reduce disease-related risks and have become a major concern for food producers and customers by positively affecting the host by increasing the viability and metabolites of the gastrointestinal microbiota [101,102]. Synbiotics also aim to improve bacterial survival, water retention, starch hydrolysis, and antibiotic resistance, as well as reduce inflammation, sugar content, fat content and calories. Therefore, polysaccharides are ideal ingredients to produce functional foods to sustain well-being and people with dietary needs [103]. Common essential non-dairy products (containing plant polysaccharides) on the market include soy, fruit juices, cereal and meat products [104]. The prebiotic dosage depends on their type, the food matrix used, the microbial physiological structure of individuals, their health condition (healthy, diabetic, hypercholesterolemic, hypertensive), gender, and age.

##### Effects of Plant Polysaccharides on the Growth of Probiotics

In general, the most prevalent genera with probiotic properties are *Bifidobacterium* and *Lactobacillus*. LAB are the dominant microorganisms in both food and beverage fermentation such as dairy, soybean, meat products, fruits, vegetables and cereal products, resulting in lactic acid being an essential metabolic product. The fermentation process includes several variables such as nutritional ingredients, microorganisms and ambient conditions, resulting in a number of distinct fermented food varieties [105]. Therefore, probiotics should have therapeutic quantities (10^6^–10^7^ CFU/mL or g) in food when consumed, be able to endure extreme conditions in the stomach and intestines (enzymes, bile acid), and be able to connect to the epithelial cells of the gut [106]. The main positive benefits of prebiotics are increasing bacterial growth, activity (primarily *Bifidobacteria* and *Lactobacillus)* and metabolites associated with health promotion. *Saccharomyces boulardii* CNCM I-745 is a particular yeast strain with probiotic characteristics, which may experience stressful situations such as mechanical shearing, cooling and freezing, osmotic pressure, and oxygen stress in ice cream production and storage. However, the addition of polysaccharides with prebiotic characteristics has shown a practical approach to maintaining the *S. boulardii* CNCM I-745 optimal durability of synbiotic ice cream production during preparation, freezing, and preservation [107]. Furthermore, prebiotics can decrease the pH level and prevent pathogens’ growth in food products (*Salmonella typhimurium*, *Clostridium perfringens*, *Escherichia Coli*, *Salmonella enteritidis*, *Campylobacter jejuni*, or *Enterobacterium ssp*) due to their ability to create undesirable conditions for their growth by synthesizing and secreting bacteriocin such as nisin [108].

**Figure 5:**
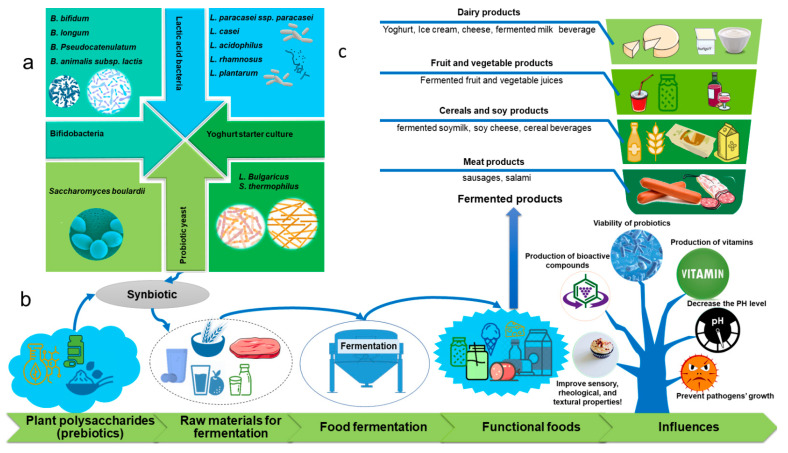
Schematic demonstration of the potential influence of plant polysaccharides in food fermentation. (**a**) probiotic bacteria most used in synbiotic fermentation, (**b**) fermentation process, (**c**) synbiotic fermented products with increased health benefits.

Table 2 below summarizes the influence of plant polysaccharides on the growth of probiotics in fermented foods, while Figure 5 represents the potential influence of plant polysaccharides in food fermentation. Dairy products are on top of probiotic food production, among which yogurt is becoming more popular than others due to its living LAB, higher digested nutrients, taste, gel-like texture, and mouthfeel [109]. During fermentation and refrigerated storage, probiotics can utilize some plant polysaccharides to grow and improve their survival rate [110]. For example, the prebiotic effect of β-glucan in yogurt improved the viability and metabolic activity of the probiotic *B. animalis subsp. lactis* strain *Bb-12* and helped maintain the probiotic levels to deliver their therapeutic effects (above 7 log CFU/g) to maintain the required level of viable cells [111]. More recently, Wang et al. [12] have reported the prebiotic effect of *Sphallerocarpus gracilis* polysaccharides (SGP) in milk fermentation. The growth of *Streptococcus thermophilus*, *Lactiplantibacillus plantarum*, and *Lacticaseibacillus rhamnosus*, as well as acidifying activity, were improved by the addition of crude SGP to milk. Fermented milk supplemented with crude SGP had a significantly greater viable probiotic population throughout shelf life than fermented milk supplied with fructooligosaccharides or inulin.

Recent research has shown that fermented soy beverages and soy cheese with synbiotics can enhance their beneficial health, texture, and taste [112]. The authors demonstrated that the lactic acid fermentation method produced synbiotic soymilk that resulted in functional foods with enhanced health advantages, such as an angiotensin-converting enzyme (ACE) inhibitory action. Furthermore, several synbiotic cereal beverages including synbiotic oat-based drinks, maize-blended rice drinks, and pomegranate drinks containing polysaccharides, mainly as prebiotics, and probiotic bacteria such as *L. acidophilus* and *Bifidobacterium* [113], were introduced and are considered as essential sources of carbohydrates, minerals, dietary fiber, protein, and vitamins in human nutrition [114]. Moreover, these beverages exhibited appropriate sensory qualities (pH, viscosity and titratable acidity) and biological activities (radical scavenging activity), with significant levels compared to unfermented drinks. Fruits and vegetables have also been focused on producing fermented non-dairy synbiotic beverages including carrot-orange juice, orange juice, hibiscus tea mixed beverages, and nectar mixed with different polysaccharide concentrations [115,116,117,118,119], and are healthy sources of antioxidants, minerals, and bioactive compounds [120]. Overall, these studies showed a strong interaction between the polysaccharides (prebiotics) and the fruit and vegetable beverage matrices. Moreover, the addition of polysaccharides can improve the growth of probiotics, thus keeping their concentration above the recommended minimum during the processing and storage of beverages, surviving gastrointestinal digestion, and reaching the intestine. For instance, during 72 h of fermentation and 30 days of refrigerated storage, apple cider, orange and grape juice fortified with either 4% long-chain or 4% short-chain inulin fiber attained a mean viable count of at least 10^7^ CFU/mL of *L. rhamnosus GR-1* [121]. Furthermore, millet, rye, and alfata sprouts were combined with probiotic bacteria and polysaccharides to produce an innovative synbiotic beverage. After 21 days, the synbiotic beverage included 10^8^ CFU/mL of *L. casei*, with excellent survival throughout the storage period (10^8^ CFU/mL) and 10^6^ CFU/mL of *L. plantarum*. Inulin and oligofructose increased strain growth and viability in cold storage while also providing better sensory scores [4].

**Table 2:** Influence of plant polysaccharides on probiotic viability in fermented food products.

Product	Polysaccharide	Probiotics	VC	Effects	References
Dairy Products					
Yogurt	inulin	*BB*, *LB*, *SS*, *LA*	6.40–7.78	Increased the organoleptic properties of low-fat synthetic yogurt and was comparable to full-fat probiotic yogurt in its performance characteristics.	[122]
		*BL*, *LA*, *ST*, *LB*	>6.0	There were appropriate sensory quality attributes and had identical ratings to the control yogurt study.	[123]
	TG, inulin	*LC*, *BB*	6.0–7.8	The texture of the yogurt was degraded, the syneresis was increased, and the sensory score was low.	[14,124]
	β-glucan	*BA*	>7.0	Sensory characteristics of probiotic yogurts enhanced hastened acidification and increased viscosity.	[111]
	SGP	*ST*, *LB*, *LPL*, *LRH*	>6.0	There was an enhancement of both the proliferation of LAB and acidifying activity.	[12]
	Inulin, modified starch	*LC*	>6.0	There was a detrimental effect on product acceptability (overall impression, flavor, appearance, and texture).	[125]
Probiotic powder milk	Hi-maize starch	*LPL*	>8.0	There were consistently maintained viable cell counts in refrigerated conditions, simulated gastric and intestinal transit.	[126]
Creamy goat cheese	Inulin	*LA*, *BA*	>6.0	Better consistency and were less firm; increased fatty acids, lactic acid and essential amino acids lead to higher acidity and lower pH.	[127]
Ice cream	Inulin	*SB*	>6.0	Enhance the physicochemical properties.	[107]
	Inulin, resistant starch	*LPL*	>7.0	There was a substantially improved probiotic viability; microcapsules containing inulin outperformed those containing starch in terms of probiotic life.	[128]
Non-dairy products					
Soymilk	Inulin	*BA*, *LA*, *ST*	>6.0	Fermentation reduced the amount of raffinose and stachyose. Therefore, there is no impact on the rate of acidification.	[129]
Fruit and vegetable Juices	Inulin	*LB*	>6.0	Fermented fig juices increased polyphenols’ bioavailability and were rich in antioxidants.	[130]
		*LPL*, *LA*	>6.0	The quality of juices increased; monosaccharide concentration remained high, and the best survival of *L. plantarum* at 30 days.	[115,116]
		*LRH*	>7.0	Overall acceptability due to flavor, texture and seemed to favor apple cider juice with long-chain inulin fiber.	[121]
Cereal beverages	Inulin	*LC*, *LPL*	>6.0	There were good sensory qualities and viability of over 55% for all the strains under gastric conditions.	[4]

VC: viability count (log CFU/mL or g); TG, Tragacanth gum; SGP, Sphallerocarpus gracilis polysaccharides; SB, Saccharomyces boulardii; BB, Bifidobacterium bifidum; LB, Lactobacillus bulgaricus; ST, Streptococcus thermophilus; LA, Lactobacillus acidophilus; BL, Bifidobacterium lactis; LP, Lactobacillus paracasei; LC, Lactobacillus casei; BA, Bifidobacterium animalis subsp. Lactis; LR, Lactobacillus reuteri; LRH, Lactobacillus rhamnosus, LF, Lactobacillus fermentum; LPL, Lactobacillus Plantarum.

##### The Health Benefits of Fermented Foods Containing Plant Polysaccharides and Probiotics

The development of functional foods is growing to help tackle public health issues, including chronic diseases such as obesity, cardiovascular disease, cancer and diabetes. Table 3 denotes the studies on the health benefits of fermented foods containing plant polysaccharides. For example, Ahmad et al. [131] demonstrated an improvement in lipid profiles, namely lowered total cholesterol, low-density cholesterol lipids, and blood pressure after hypercholesterolemic individuals were given yogurt from sheep and cows combined with dietary fiber for 30 days. Ice cream combined with water-soluble extracts from rice by-products and prebiotic ingredients had a better health index such as improved ACE inhibitory activity and antioxidant activity [132]. A synbiotic yogurt demonstrated significant hypolipidemic potential through a weekly biological analysis for lipids profile of rabbits with hyperlipidemia after feeding a diet containing varying quantities of synbiotic yogurt. The total triglyceride levels (155.00 ± 8.88 mg/dL), cholesterol levels (124.00 ± 7.10 mg/dL), low-density lipoprotein levels (13.27 ± 0.76 mg/dL), and extremely low-density lipoprotein levels (57.04 ± 3.27 mg/dL) decreased significantly, while high-density lipoprotein levels (53.70 ± 0.35 mg/dL) improved [133].

Another study on yogurt consumption with a Korean citrus hallabong peel polysaccharide (RG) showed that yogurt’s daily consumption with RG improved natural killer (NK) cells’ potential and attenuated the levels of pro-inflammatory cytokines. After 8 weeks of therapy, the testing group showed considerable decreases in IL-6 and IL-1 levels compared to the placebo group. These results highlight the possible use of RG yogurt in nutritional supplements as a route to improve immune efficiency and decrease chronic inflammation [134]. As a result, adding prebiotic polysaccharides to fermented materials would boost the products’ anti-hypertensive and anti-diabetic characteristics studied in vitro.

**Table 3:** Health benefits of the intake of fermented foods with plant polysaccharides.

Products	Polysaccharide	Health Effect	Condition	References
Yogurt	Dietary fiber	Fortified yogurts substantially lowered TC, LDL-C, and blood pressure in hyper cholesterol patients.	In vivo	[131,135]
	Inulin	Synbiotic yogurt’s intake strengthened hepatic characteristics in nonalcoholic fatty liver disease patients.	In vivo	[136]
	Inulin and wheat fiber	There was a rise in proteolysis levels resulting in antioxidant and ACE-inhibitory properties	In vitro	[137]
	PR	It intensified the levels of pro-inflammatory cytokines and improved the activity of NK cells.	In vitro	[134]
Sheep milk ice cream	Inulin	*L. casei* had a strong adhesive capacity for Caco-2 cells, ensuring a therapeutic impact on the host; significant antioxidant and anti-hypertensive properties increased the product’s bioactivity.	In vitro	[138]
		Synbiotic group significantly reduced p53 expression and apoptosis index in colonic crypts and significantly reduced micronucleated colon cells.	In vivo	[139]
Soy yogurt	Inulin	There was a protective effect on milk cultures’ viability, with decreased pH, total phenolic content and increased acidity had higher antioxidant activity during storage.	In vitro	[112]
Salami	Dietary fibers	There was an increase in antioxidant capacity, production of SCFAs, the change in gut microbiota structure, and reduction of intestinal pathogens.	In vitro	[140]
	Citrus fiber	In four weeks, there were improved inflammatory, immunological, antioxidant plasmatic markers, and butyrate production.	In vivo	[141]
Sausages	Inulin	There was an influence on the intestinal microbiota activity, elevated levels of SCFAs in fecal and plasma metabolome, and increased *Bifidobacterium*.	In vivo	[142]
Oat-Banana Fermented Beverage	β -glucan	The relative gene expression levels in the selected strains were related to the L-lactic acid produced in the two media. The plant matrix promoted greater ldhL gene expression in the first 4 h of the experiment.	In vitro	[143]

TC, Total cholesterol; LDL-C, Low-density lipids cholesterol; ACE, Angiotensin-converting enzyme; SCFAs, Short-chain fatty acids; NK, Natural killer; PR, Polysaccharide *rhamnogalacturonan;* ldhL, L-lactate dehydrogenase gene.

#### 3.2.2. Plant Polysaccharides and Microbial Fermentation Products as Sources of Bioactive Compounds

Fermentation can produce or increase bioactive compounds’ bioavailability [6] and generate new substances including short-chain fatty acids (SCFAs), B-group vitamins, organic acids, microbial polysaccharides, ethanol and bacteriocins, with specific functions such as nutritional supplements, weight loss supplements, and dietary substitutes [6]. The SCFAs (butyrate, propionate and acetate) are the main components of fermentation products and are easily absorbed by the human body [144], and provide a variety of health advantages for the host, including immune control [145], regulation of mucosal inflammation, proliferation, mineral absorption, colorectal carcinogenesis, and nitrogen compound removal [146].

Asarat et al. [147] collected SCFAs from fermented reconstituted skim milk with polysaccharides (β-glucan, inulin or resistant starch/hi-maize). It was found that, compared with β-glucan or hi-maize, inulin significantly promoted SCFAs production by *L. rhamnosus*. In addition, SCFAs extracts were subsequently used in human peripheral blood mononuclear cells (PBMCs) in vitro immune modulation trials. SCFAs inhibited the expression of tumor necrosis factor-alpha, interleukin (IL)-12, interferon-gamma (IFN-g), and transforming growth factor beta-1 (TGF-b1) in lipopolysaccharide LPS-stimulated PBMCs, but they increased the expression of IL-4 and IL-10. The study showed that SCFAs regulated anti-inflammatory cytokines media in LPS-stimulated PBMCs.

*Mangefira pajang* polysaccharides and inulin improved the growth of probiotics and their capacity to generate organic acids (propionic, lactic and acetic acids) during probiotic yogurt production, but there was no significant difference in organic acid production between inulin yogurt and *Mangefira pajang* polysaccharide yogurt [148]. Plant polysaccharides as prebiotics can enhance the growth of selected bacteria and increase natural vitamin levels during the fermentation of both dairy and non-dairy products. Albuquerque et al. [8] assessed the effect of fructooligosaccharide (FOS) and passion fruit by-products on the fermentation of different soymilk formulations through increasing folate content using LAB. *St. thermophilus* provided the most abundant folate in all products, whether used alone or in combination with *lactobacilli* strains. The findings were viewed as a less costly technical method to increase folate in non-dairy fermented foods. Regarding folate supplements, are mainly synthetic folate form (folic acid), which is synthesized chemically. More than 60 countries worldwide are undertaking mandatory folate fortification programs that rely on these synthetic vitamins to reduce the incidence of neural tube defects [149]. Consequently, high folic acid consumption was linked to several negative side effects, including hiding symptoms of vitamin B12 insufficiency and potentially promoting the development of colorectal cancer, while folates naturally found in foods or synthesized by probiotics and prebiotics do not have these detrimental effects due to its relatively low absorption rate [150].

## 4. Conclusions and Future Perspectives

Plant polysaccharides are used in fermented foods such as dairy products (fermented milk, yogurt, ice cream, and cheese), fruits and vegetables, soy and cereal products, as well as meat products due to their functionalities and biological activities. Their suitability in terms of improving food properties (acceptability, flavor, appearance, and texture), stimulating the growth of probiotic bacteria (LAB and Bifidobacteria), and helping to strengthen the gastrointestinal and immune systems, primarily as prebiotics, with promising results for anti-hypertensive and anti-diabetic properties, increased blood pressure lipid composition, and enhancement of ACE inhibitory activity, was proven in vivo or in vitro.

Despite tremendous advances in plant polysaccharide research in fermented foods, there are still more challenges in practical applications. Foremost, extraction is a crucial step in producing usable plant polysaccharides. Their purity and extraction rates have improved from one method to another; nevertheless, specific methods are unfriendly to the environment, time-consuming and require high temperature, resulting in low polysaccharide yield and quality. Since obtaining pure polysaccharides with a single approach is inefficient, combining techniques is necessary to maintain the yield, consistency, chemical structure, and biological activity of polysaccharides. Moreover, it is essential to produce high-purity polysaccharides on a wide scale to satisfy the requirements as processed food ingredients or additives while maintaining their structural stabilities and bioactivities.

The next consideration is meat products, which accentuate the negative features of saturated fat, excessive salt, chemicals, and lack of fiber in consumer diets. Therefore, adding polysaccharides and probiotic strains to fermented meat products has proven beneficial. More research is required on using plant polysaccharides (prebiotics) in meat products to improve nutritional qualities due to their potential effects and no side effects on human health. Innovative formulations with increased polysaccharides and probiotics and lower salt, nitrite/nitrate, or cholesterol content may enhance meat product technology and result in more nutritional fermented meat products.

Furthermore, people nowadays are increasingly interested in consuming nutritious foods that can improve their lives. Another area of interest for future research of plant polysaccharides is determining their synbiotic advantages with probiotics in producing biological components such as vitamins (folate), enzymes (β-glycosidase), and other compounds in fermented dairy and non-dairy products. For example, more than 60 countries have adopted mandatory folic acid fortification programs, which are chemically synthesized, and high doses of folic acid intake were associated with several adverse effects, including hiding symptoms of vitamin B12 deficiency and the possibility of colon cancer. Natural folate production by combining polysaccharides and lactic acid bacteria in fermented milk is a promising approach to increase folates content in foods. In a word, given the prevalence of non-communicable diseases, functional foods containing plant polysaccharides are the most unique approach to preventive or complementary therapies. In this review, we found few studies on the therapeutic effects of plant polysaccharides in fermented foods. Therefore, additional clinical trials are needed to evaluate whether the benefits of these foods will be sustained in longer-term treatments.

## Acronyms Used

LAB	Lactic acid bacteria	UHPE	Ultra-high pressure extraction
MW	Molecular weight	TC	Total cholesterol
FOS	Fructooligosaccharide	LDL-C	Low-density lipids cholesterol
SCFAs	Short-chain fatty acids	ACE	Angiotensin-converting enzyme
UAE	Ultrasonic-assisted extraction	PR	Polysaccharide *rhamnogalacturonan*
ASE	Alkaline solvent extraction	OP	Okra polysaccharide
EAE	Enzymatic-assisted extraction	KP	Kernel polysaccharide
UAEE	Ultrasonic-assisted enzymatic extraction	DOP	*Dendrobium Officinale* polysaccharide
MAE	Microwave-assisted extraction	TG	*Tragacanth* gum
UMAE	Ultrasound-microwave assisted extraction	ldhL	L-lactate dehydrogenase gene
HPLC	High-performance liquid chromatography	PBMCs	peripheral blood mononuclear cells
T2DM	type 2 diabetes mellitus		
